# Supplementary material for: Viral outbreaks involve destabilized evolutionary networks: evidence from Ebola, Influenza and Zika
Source: Sci Rep. 2017 Sep 19;7:11881. doi: 10.1038/s41598-017-12268-9 (PMC5605547; doi:10.1038/s41598-017-12268-9)
Supplement: Supplementary file 1 — Supplementary Information [file 41598_2017_12268_MOESM1_ESM.pdf]

Supplementary Information: Viral outbreaks involve destabilized evolutionary networks: evidence from Ebola, Influenza and Zika

Stéphane Aris-Brosou[1,2,⋆], Neke Ibeh[1] and Jessica Noël[1]

[1]Department of Biology, University of Ottawa, Ottawa, ON K1N 6N5, Canada

[2]Department of Mathematics and Statistics, University of Ottawa, Ottawa, ON K1N 6N5, Canada

[⋆]Correspondence: sarisbro@uottawa.ca

Table S1. Alignments used in this study. For each virus are shown the genes that were analyzed, the epidemiological status (NP: non-pandemic/non-outbreak years; PDM: pandemic/outbreak years), the sampling years, and the number of sequences in each alignment (N_{seq}).

Virus	Gene	Epidemiology	Years	N_{seq}
Ebola	GP	NP	1976-2014	66
		PDM	2014-2015	1023
Zika	NS5	NP	1947-2014	79
		PDM	2014-2015	82
Influenza	HA	NP	2008-2009	452
		PDM	2009	1186
	NA	NP	2009	434
		PDM	2009	656
	HA	PDM	2009-2010	2442
		PDM	2010-2011	778
		PDM	2011-2012	243
		PDM	2012-2013	438
		PDM	2013-2014	741
		PDM	2014-2015	77
		PDM	2015-2016	95
	NA	PDM	2009-2010	1495
		PDM	2010-2011	646
		PDM	2011-2012	175
		PDM	2012-2013	305
		PDM	2013-2014	433
		PDM	2014-2015	56
		PDM	2015-2016	56

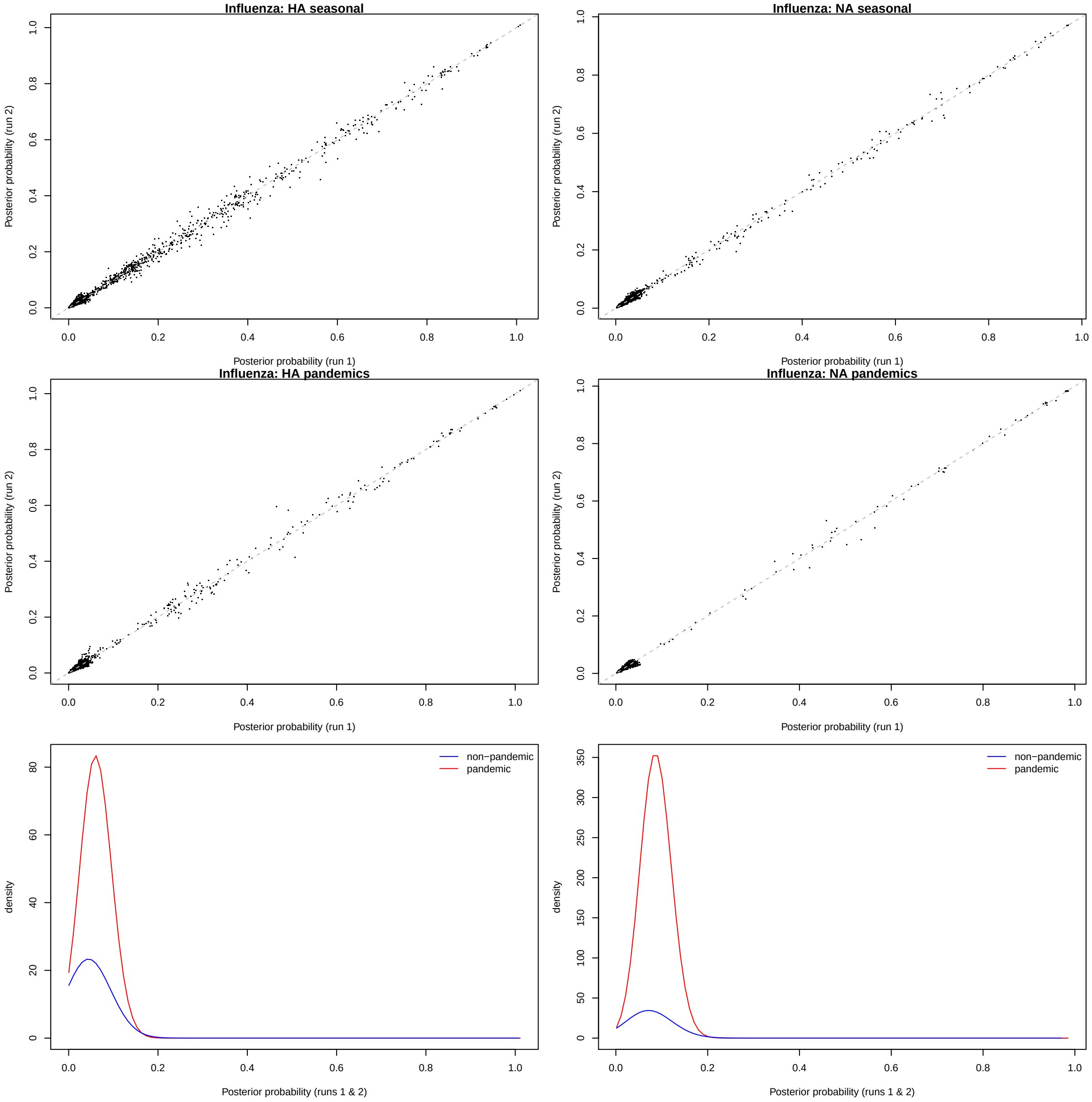

Figure S1. **Convergence of the BGM analyses for the 2009 Influenza data.** Results of two independent MCMC runs are shown for HA (left) and NA (right) for estimated posterior probabilities of correlated evolution for seasonal (top) and pandemic (middle) viruses. Plots in the bottom row show the distribution of posterior means, showing that most pairs of sites exhibit weak interactions for both seasonal (blue) and pandemic (red) viruses.

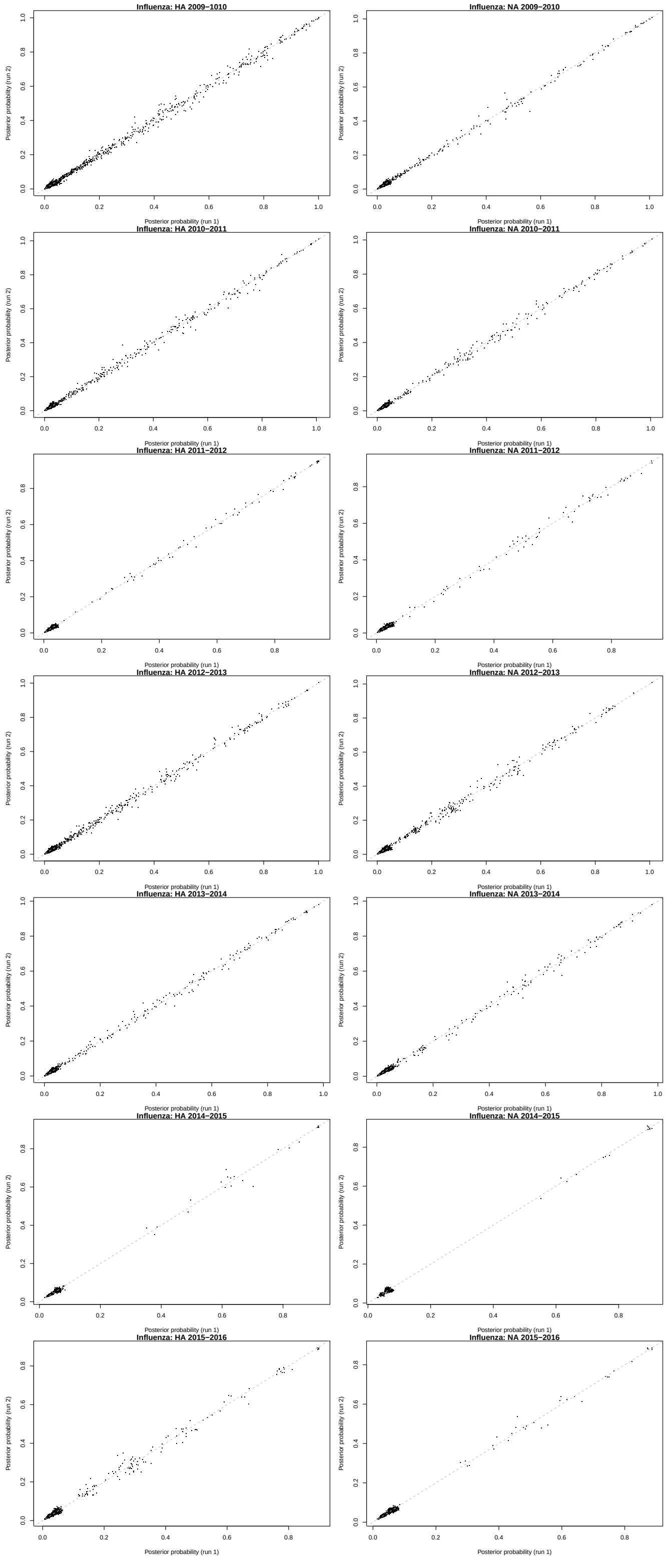

Figure S2. **Convergence of the BGM analyses for the 2009-2016 Influenza data.** Results of two independent MCMC runs are shown for HA (left) and NA (right) for estimated posterior probabilities of correlated evolution. Rows are ordered from the 2009-2010 to the 2015-1016 seasons.

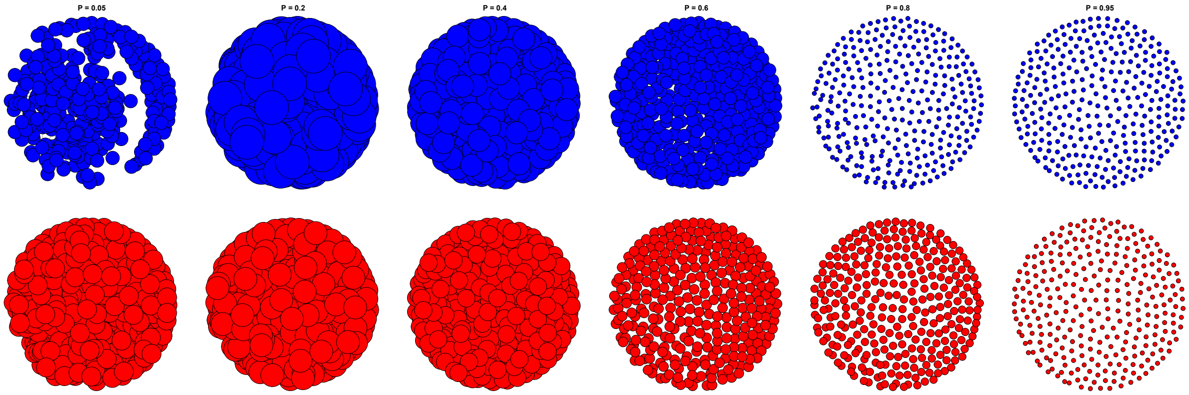

Figure S3. Correlation network of seasonal and pandemic Influenza viruses in 2009 for the hemagglutinin protein (HA). Networks of correlated amino acids in the HA gene are shown in each panel. The top row shows networks for the viruses circulating before the 2009 pandemic (blue); the bottom row shows networks for pandemic viruses (red). Each column shows networks for different strengths of correlation, from weak ($P = 0.05$) to strong ($P = 0.95$).

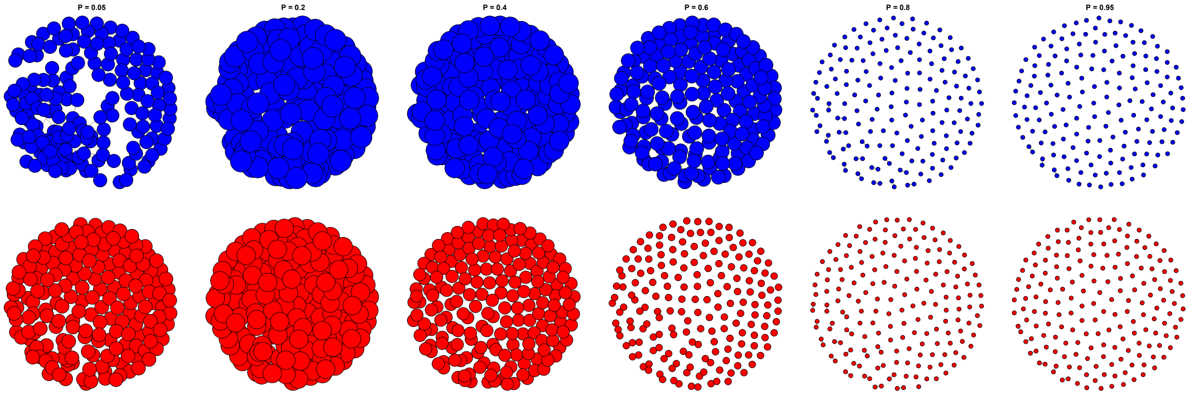

Figure S4. Correlation network of seasonal and pandemic Influenza viruses in 2009 for the neuraminidase protein (NA). Networks of correlated amino acids in the NA gene are shown in each panel. The top row shows networks for the viruses circulating before the 2009 pandemic (blue); the bottom row shows networks for pandemic viruses (red). Each column shows networks for different strengths of correlation, from weak ($P = 0.05$) to strong ($P = 0.95$).

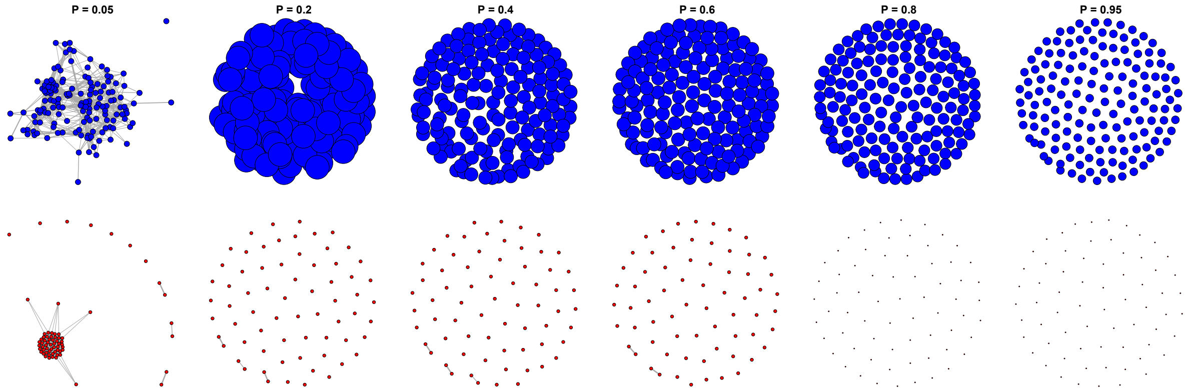

Figure S5. **Correlation network of pre-outbreak and outbreak Zika viruses.** Networks of correlated amino acids in the NS5 gene are shown in each panel. The top row shows networks for the viruses circulating before the 2014 outbreak (blue); the bottom row shows networks for outbreak viruses (red). Each column shows networks for different strengths of correlation, from weak ($P = 0.05$) to strong ($P = 0.95$).

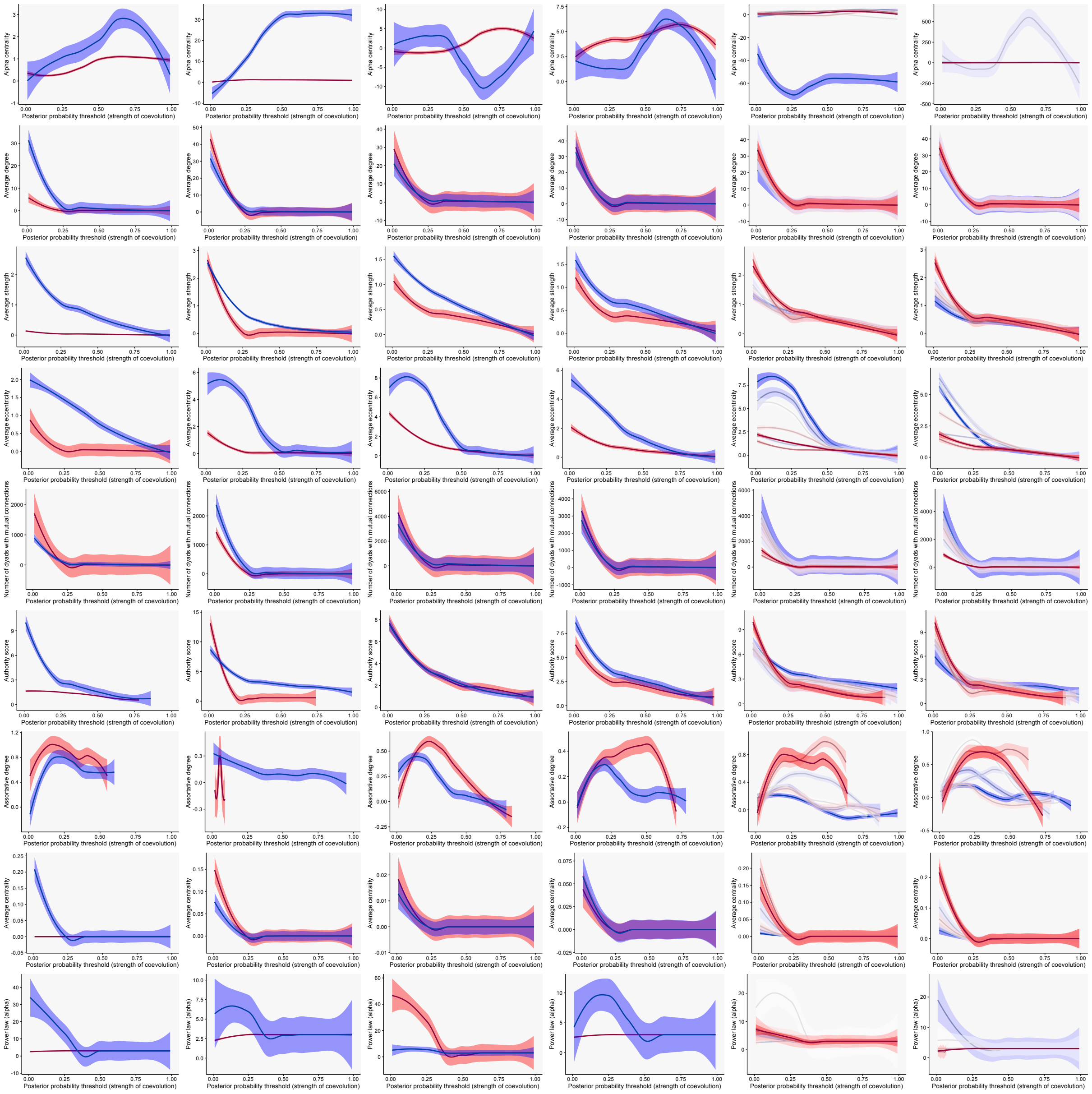

Figure S6. Network properties between pandemic and non-pandemic viruses. Results are shown for Ebola (column 1), Zika (2) and Influenza viruses (for HA and NA circulating in 2009 in (3) and (4), respectively, and for pandemic viruses circulating since then, season by season (5-6)). Pandemic viruses are show in red, while non-pandemic one are in blue. Shading: 95% confidence envelopes of the LOESS regressions.

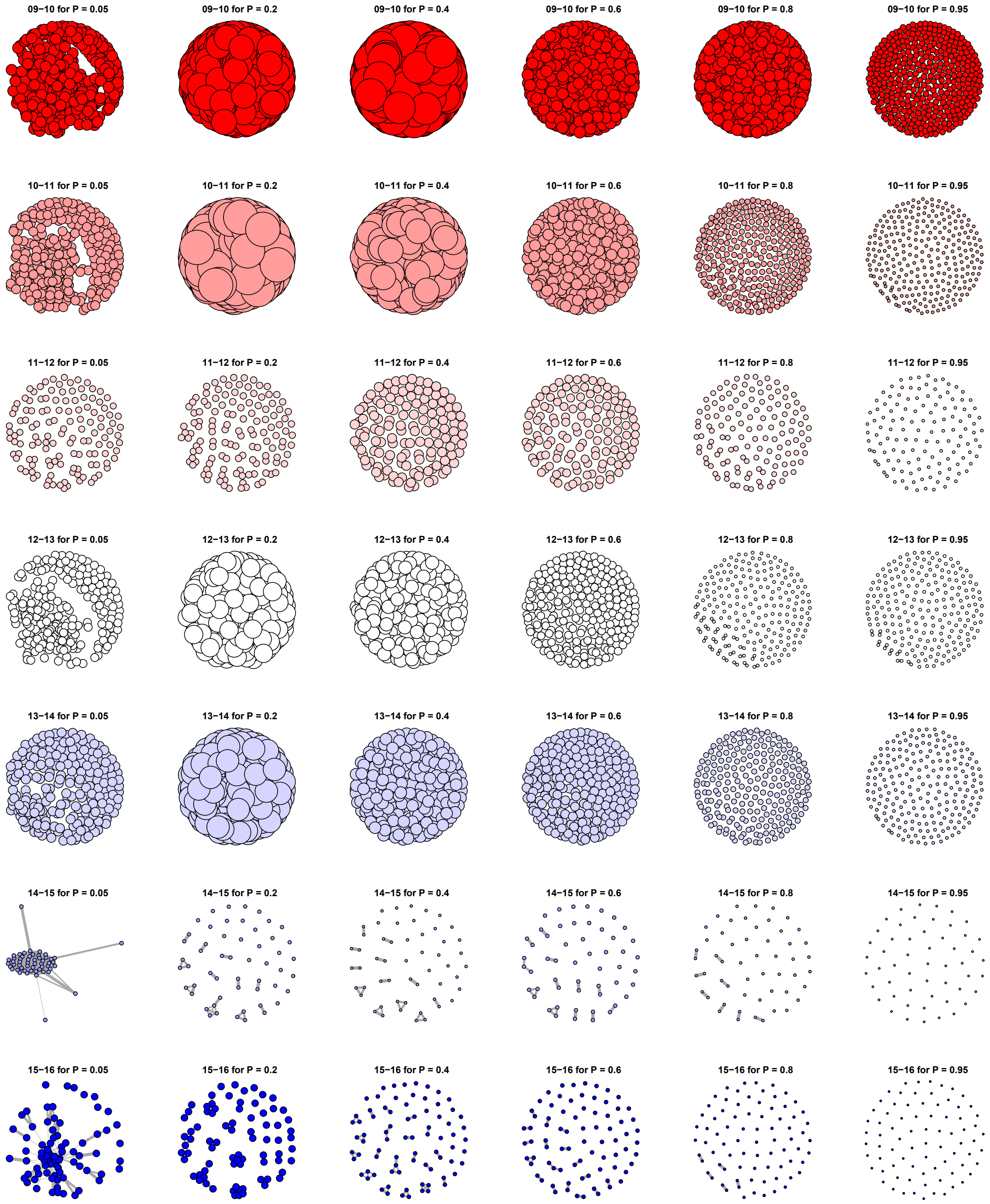

Figure S7. Correlation network of Influenza viruses between the 2009-2010 to 2015-2016 seasons. Networks of correlated amino acids in the HA gene are shown in each panel. Seasons are color-coded from warm (pandemic) to cold (non-pandemic) seasons.

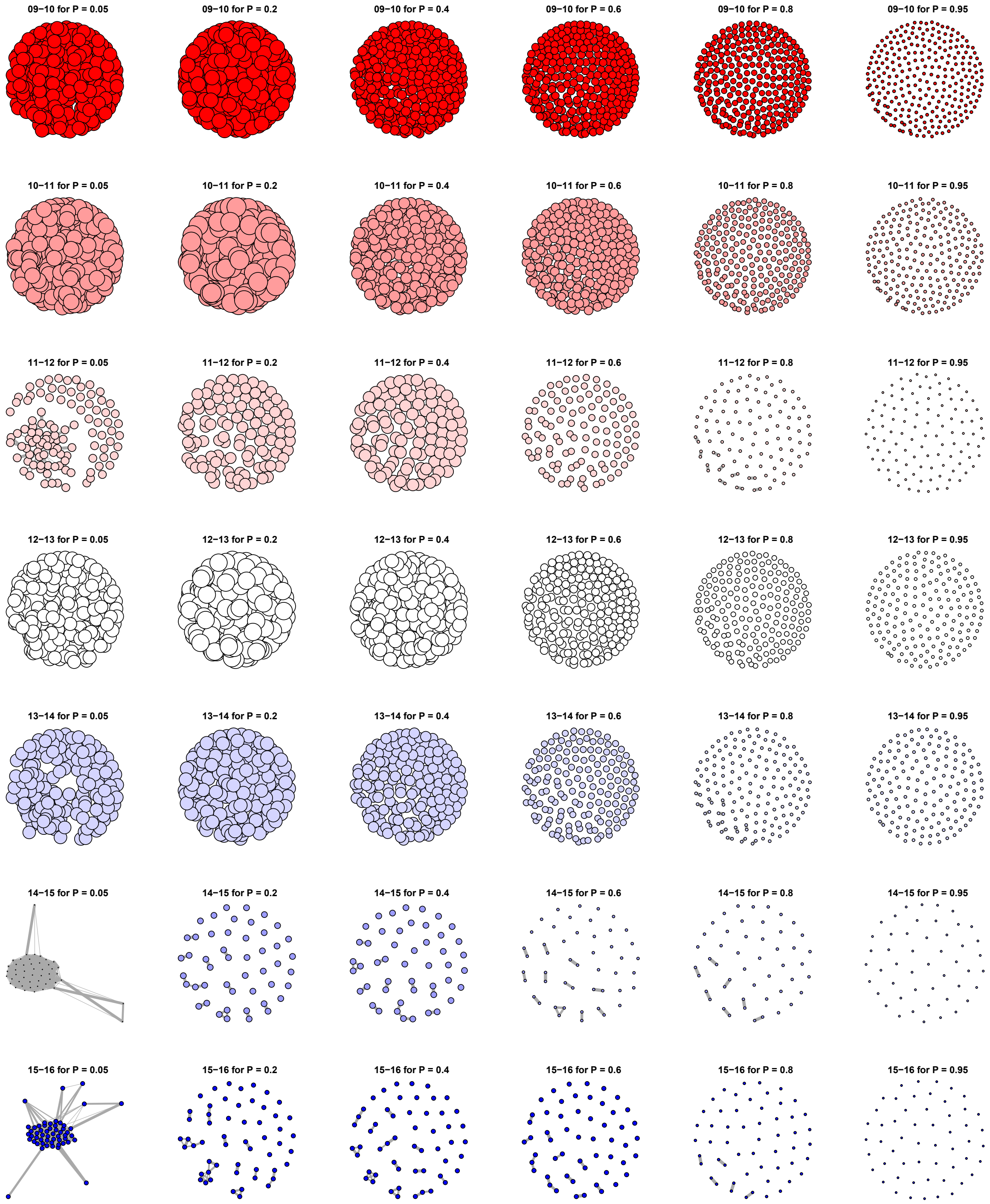

Figure S8. Correlation network of Influenza viruses between the 2009-2010 to 2015-2016 seasons. Networks of correlated amino acids in the NA gene are shown in each panel. Seasons are color-coded from warm (pandemic) to cold (non-pandemic) seasons.
